# Effects of Resistance Exercise and Whey Protein Supplementation on Irisin Levels in Patients with MASLD Under a Calorie-Restricted Diet

**DOI:** 10.3390/nu18081272

**Published:** 2026-04-17

**Authors:** Feng-Rui Zhang, Chae-Been Kim, Dohyun Ahn, Jinwoo Sung, Ju-Hwan Oh, Hae-Ri Heo, Eun-Ah Jo, Hong-Soo Kim, Jung-Jun Park

**Affiliations:** 1 Department of Sport Science, Pusan National University, Busan 46241, Republic of Korea; zfree@pusan.ac.kr (F.-R.Z.); chaebeen@pusan.ac.kr (C.-B.K.); ahndo99@pusan.ac.kr (D.A.); sjw920412@pusan.ac.kr (J.S.); mtoto0417@pusan.ac.kr (J.-H.O.); heohaeri@pusan.ac.kr (H.-R.H.); jea0543@pusan.ac.kr (E.-A.J.); 2 Research Institute of Human Ecology, Pusan National University, Busan 46241, Republic of Korea; 3 Department of Kinesiology and Sport Management, Texas Tech University, Lubbock, TX 79409, USA; 4 Gastroenterology, Soon Chun Hyang University Cheonan Hospital, Cheonan 31151, Republic of Korea; khskhs@sch.ac.kr

**Keywords:** controlled attenuation parameter (CAP), irisin, metabolic dysfunction-associated steatotic liver disease (MASLD), resistance exercise, whey protein supplementation

## Abstract

**Objectives**: The aim of this study was to explore the combined effects of resistance exercise and whey protein supplementation on plasma irisin levels in patients with metabolic dysfunction-associated steatotic liver disease (MASLD) under a 30% calorie-restricted weight loss diet. **Methods**: Thirty adult patients with MASLD were randomized into the following three groups for a 4-week intervention: calorie restriction group (CR) (*n* = 8), CR with resistance exercise group (EX) (*n* = 11), and CR with resistance exercise and whey protein group (EX + P) (*n =* 11; 0.7 g/kg per day). All participants received boxed meals providing 70% of their total energy expenditure. The participants in the resistance exercise groups performed full-body resistance exercises 5 days/week (50–75% one-repetition maximum). Plasma irisin level, controlled attenuation parameter (CAP), and body composition were assessed before and after the intervention. **Results**: Plasma irisin levels significantly increased in the EX (+2.24 ng/mL, *p* = 0.016) and EX + P (+4.86 ng/mL, *p* = 0.004) groups but not in the CR group. Muscle mass increased significantly only in the EX + P group. The CAP decreased in all groups. The change in irisin level was negatively correlated with the change in CAP (r = −0.459, *p* = 0.032). **Conclusions**: Resistance exercise under calorie-restricted conditions effectively increased plasma irisin levels in patients with MASLD, whereas caloric restriction alone did not. Furthermore, a stronger increasing trend in the plasma irisin levels was observed with whey protein supplementation. An increase in irisin levels was significantly associated with hepatic fat reduction, suggesting that irisin may serve as a biomarker reflecting improvements in hepatic steatosis following lifestyle intervention.

## 1. Introduction

Metabolic dysfunction-associated steatotic liver disease (MASLD) is the newly proposed nomenclature that replaces the term non-alcoholic fatty liver disease. MASLD is characterized by excessive hepatic triglyceride accumulation in the presence of at least one cardiometabolic risk factor [1,2]. MASLD is now recognized as the most common chronic liver disease worldwide, affecting over 30% of the adult population [3].

According to guidelines from the European Association for the Study of the Liver and others, sustained weight loss of 3–5% through dietary and behavioral interventions can effectively reduce intrahepatic fat accumulation [1,4]. Specifically, a reduction in daily caloric intake by at least 30%, or approximately 750–1000 kcal, can significantly decrease liver fat content [5]. Notably, calorie-restricted diets reduce not only body weight and fat mass but also skeletal muscle mass [6]. Sarcopenia and MASLD share overlapping risk factors, pathophysiological mechanisms, and etiological aspects [7]. Incorporating resistance exercise into a calorie-restricted diet improves muscle mass and muscle function [8]. Therefore, it is necessary to incorporate resistance exercise alongside a calorie-restricted diet to improve MASLD.

During exercise, skeletal muscles release myokines that contribute to metabolic homeostasis [9]. Among these, irisin—a contraction-induced myokine [10]—promotes the browning of white adipose tissue, thereby increasing energy expenditure. It has also been linked to systemic energy regulation and metabolic disorders [11]. In addition, it reduces the production of reactive oxygen species (ROS) through multiple mechanisms, such as upregulating autophagy while downregulating endoplasmic reticulum stress, inflammasome activation, and cell death, thereby protecting hepatocytes from oxidative stress-induced damage [12]. Patients with MASLD generally exhibit lower circulating irisin levels compared to those in healthy controls [13,14]. Resistance exercise has been shown to increase circulating irisin [15], whereas findings under caloric restriction alone are inconsistent [16,17], highlighting the necessity to distinguish the effects of contractile stimuli from those of energy deficit.

Furthermore, protein supplementation can enhance the effects of resistance exercise [18] and has been suggested to contribute to more rapid reductions in hepatic fat than resistance exercise alone [19]. However, how these three interventions—caloric restriction, resistance exercise, and protein supplementation—interact to regulate circulating irisin in the MASLD population is unknown. This limits the optimization of clinical intervention strategies for MASLD.

Therefore, this study aimed to investigate the combined effects of resistance exercise and whey protein supplementation on plasma irisin levels in patients with MASLD under a 30% calorie-restricted weight loss diet. In addition, we further examined whether changes in irisin are associated with reductions in hepatic fat to explore the scientific evidence for exercise and nutritional intervention strategies for MASLD.

## 2. Materials and Methods

### 2.1. Participants

Thirty adult participants were recruited through advertisements posted at community centers, universities, and online platforms. Interested individuals were screened through one-on-one interviews with the research team to confirm eligibility. Inclusion criteria were as follows: male or female aged 20–60 years diagnosed with MASLD based on clinical diagnostic guidelines; no engagement in regular exercise within the past 3 months (defined as exercising less than once per week for <30 min each session); clearance for exercise participation based on the Physical Activity Readiness Questionnaire; no significant weight change in the past 3 months; and a body mass index of ≥23.0 kg/m^2^. The exclusion criteria were as follows: allergy or intolerance to milk or dairy products; current smoking; pregnancy or lactation; or a diagnosis of heart or kidney disease.

This study was approved by the Institutional Review Board of the Pusan National University (IRB No. 2023_229_HR; approval date: 26 December 2023) and preregistered with the Clinical Research Information Service (KCT0009143; registration date: 30 January 2024) prior to first participant enrollment. All participants provided written informed consent before participation.

### 2.2. Study Design

**Figure 1:**
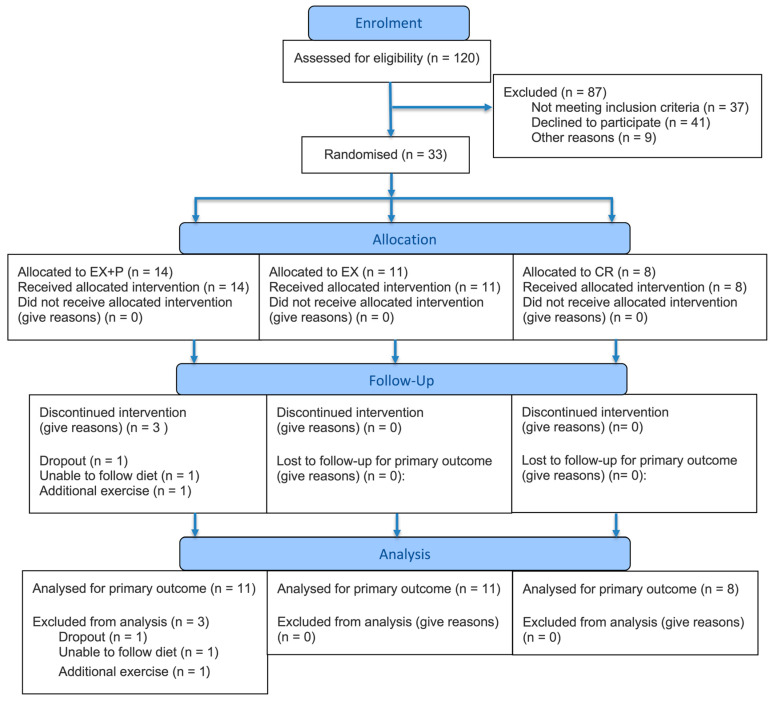
Flow diagram of participant progression through the study.

This randomized, controlled, partially double-blind, placebo-controlled trial involved a 4-week intervention period. A computer-generated randomization sequence (simple randomization) was created by a researcher independent of outcome assessment and intervention implementation. The participants were randomly assigned to one of the following groups: calorie restriction alone with placebo supplementation (CR; placebo control), calorie restriction with resistance exercise and placebo supplementation (EX), and calorie restriction with resistance exercise and whey protein supplementation (EX + P). The participant flow is summarized in Figure 1. Both the participants and research staff were blinded to the supplementation type (whey protein or placebo), which was packaged and coded by an independent third party.

All participants underwent standardized pre- and post-intervention assessments. On day 1 of assessment, the participants completed a physical activity questionnaire to assess baseline activity levels, followed by measurements of body composition, resting metabolic rate (RMR), intrahepatic fat content, and venous blood sampling. All assessments were conducted after an overnight fast of 8 h. During fasting, the participants were instructed to abstain from food, beverages, alcohol, caffeine, and any substances that might affect body composition measurements. On day 2 of assessment, the participants performed a one-repetition maximum (1-RM) strength test. Throughout the 4-week intervention period, all participants received calorie-adjusted boxed meals for breakfast, lunch, and dinner. These meals were designed by the research team based on each individual’s RMR, as measured during pre-intervention assessment. Total caloric intake was adjusted to account for the energy content of the supplements.

### 2.3. Dietary Control and Whey Protein Supplement or Placebo Intake

During the intervention period, total energy intake was strictly controlled for all participants. The total energy expenditure (TEE) for each participant was estimated by multiplying their measured RMR by a physical activity level factor [20]. Based on this, participants received three boxed meals per day, with a 30% reduction in the total caloric content to induce a calorie deficit. The caloric content of the supplements (whey protein or placebo) was subtracted to ensure precise energy control.

The total energy distribution was as follows: dietary fat accounted for 25% of total energy intake, protein intake was set at 0.8 g/kg body weight (all derived from food), and the remaining energy was provided by carbohydrates. Participants in the EX + P group received five additional daily servings of whey protein supplementation in addition to the three meals, resulting in a total daily protein intake of 1.5 g/kg body weight (0.8 g/kg from food and 0.7 g/kg from the supplement). Participants in the EX and CR groups received an isocaloric carbohydrate placebo supplement. Whey protein and placebo were provided in powder form (Maeil Health Nutrition, Pyeongtaek, Republic of Korea) and packaged in an indistinguishable manner to maintain blinding. To monitor dietary compliance, participants were instructed to record and submit video recordings of each meal to the research team. Participants were instructed to consume only the provided meals; any deviation from the prescribed diet was required to be recorded and reported immediately to the research team.

### 2.4. Resistance Exercise Program

As shown in Table 1, participants in both the EX and EX + P groups performed resistance exercise five times a week for 4 weeks. Exercise intensity ranged from 50% to 75% of the participant’s 1-RM. Each 60 min exercise session comprised a 10 min warm-up, 40 min of resistance training, and a 10 min cool-down. The main exercise program targeted the chest, back, and lower limbs, each trained twice weekly. Participants performed four sets of 8–12 repetitions per exercise, with 1–2 min of rest between sets.

### 2.5. Measurements

#### 2.5.1. Body Composition

Participants arrived at the laboratory in the morning after fasting for ≥8 h, abstaining from caffeine for ≥12 h, and avoiding alcohol and vigorous exercise for ≥24 h; they voided prior to testing. Body composition was assessed using bioelectrical impedance analysis (BIA) with the In-Body BWA 2.0 device (InBody Co., Ltd., Seoul, Republic of Korea). The measured variables included body fat percentage (%), fat mass (kg), and skeletal muscle mass (kg). Body weight was measured using a body composition analyzer (InBody 620; InBody Co., Ltd., Seoul, Republic of Korea). Height and weight were measured with the participant standing barefoot on the measurement platform, upright and facing forward. Measurements were recorded to the nearest 0.1 cm. Two consecutive measurements were obtained by the same trained assessor, and the mean value was used for analysis.

#### 2.5.2. RMR

RMR was measured using a breath-by-breath metabolic gas analyzer (Quark b^2^, COSMED, Albano Laziale, Italy). Upon arrival at the laboratory after an overnight fast of ≥8 h, participants rested quietly for 15 min. They were then positioned supine with their heads under a ventilated canopy hood, and gas exchange was recorded for 15 min while they remained awake and at rest; the first 5 min were discarded for stabilization, and the subsequent 10 min were used to calculate RMR using the Weir equation. The system was calibrated according to the manufacturer’s instructions before each testing session.

#### 2.5.3. Hepatic Fat Content

Hepatic fat content was measured using a transient elastography device (FibroScan^®^ 502, Echosens, Paris, France). During the measurement, positioned supine with the right arm raised above the head to expose the right intercostal space. The ultrasound probe was placed between the right ribs, and the controlled attenuation parameter (CAP) was recorded as the primary outcome measure.

#### 2.5.4. Blood Biochemistry Assay

After overnight fasting for 8 h, 8 mL of venous blood was drawn from the antecubital vein. Blood samples were centrifuged at 4 °C for 10 min at 3200 rpm. Plasma was separated and stored at −70 °C until analysis. Plasma irisin levels were determined using a commercially available enzyme-linked immunosorbent assay kit (cat#: DY9420-05, R&D Systems, Minneapolis, MN, USA). ELISA calibration was performed using a standard curve. Serial dilutions of the provided standards (0.25–8 ng/mL) were prepared, and optical density (OD) values were fitted using a four-parameter logistic (4PL) model. The resulting standard curve showed a good fit (R^2^ = 0.998). All standards were measured in duplicate, and sample concentrations were calculated based on the standard curve. All procedures were performed according to the manufacturer’s instructions. The intra-assay coefficient of variation (CV) was 0.081%.

### 2.6. Data Analysis

Data were analyzed using SPSS version 29.0 (IBM Corporation, Armonk, NY, USA). The Shapiro–Wilk test was used to assess the normality of the data distribution. Non-parametric statistical methods were applied to non-normally distributed variables. Participant demographics were compared using the Kruskal–Wallis H test, within-group comparisons were conducted using the Wilcoxon signed-rank test, and between-group comparisons were performed using Quade’s nonparametric ANCOVA. Parametric statistical methods were applied for normally distributed variables, including paired *t*-tests for within-group comparisons and one-way ANCOVA for participant demographics and between-group comparisons. Differences in sex distribution among the three groups were assessed using the Chi-square test. Age was included as a covariate in the ANCOVA model for between-group comparisons. Spearman’s correlation analysis was used to assess the associations between the variables. To further control for the potential confounding effect of age in correlation analyses, Spearman’s correlation analysis was conducted on the residuals after regressing each variable on age. Statistical significance was set at *p* = 0.05.

## 3. Results

### 3.1. Participant Demographics

Thirty-three participants were enrolled in this study. However, in the EX + P group, one dropped out, one was unable to follow the dietary intervention because of work obligations, and one performed additional exercises before the post-test. As shown in Table 2, the final analysis included 30 participants, including 11, 11, and 8 participants in the EX + P, EX, and CR groups, respectively. The mean ages were 33.18 ± 10.55, 32.82 ± 14.28, and 29.00 ± 7.43 years in the EX + P, EX, and CR groups, respectively. The mean body weights were 77.61 ± 12.37, 81.70 ± 8.77, and 74.88 ± 13.12 kg in the EX + P, EX, and CR groups, respectively. No statistically significant differences were observed in age, body weight or BMI, as well as sex distribution among the three groups.

### 3.2. Irisin Levels

**Figure 2:**
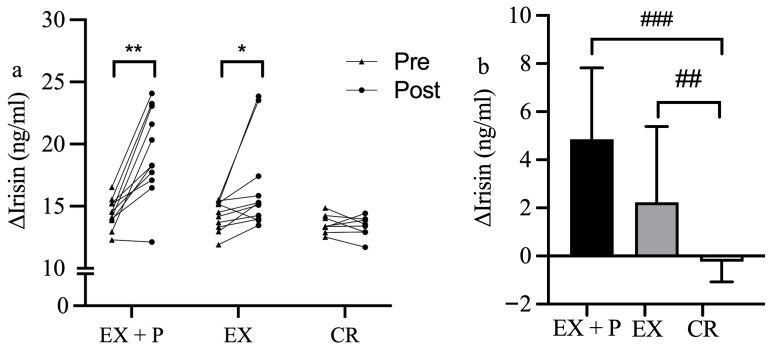
(**a**) Changes in plasma irisin levels from pre- to post-intervention in each group (EX + P, EX, and CR). ** *p* < 0.01, * *p* < 0.05 significant difference within groups. (**b**) Comparison of changes in plasma irisin levels among three groups. ### *p* < 0.001, ## *p* < 0.01 significant difference between groups.

Changes in plasma irisin after the 4-week intervention are shown in Figure 2. Plasma irisin levels significantly increased in both the EX + P group (Δ = 4.86 ± 2.97 ng/mL [from 14.45 ± 1.20 to 19.30 ± 3.58, median difference = 5.050 ng/mL, 95% CI: 2.523 to 7.516, r = 0.858, *p* = 0.004]) and the EX group (Δ = 2.24 ± 3.15 ng/mL [from 14.29 ± 1.19 to 16.53 ± 3.70, median difference = 1.424 ng/mL, 95% CI: 0.511 to 4.688, r = 0.724, *p* = 0.016]), whereas no significant change was observed in the CR group (Δ = −0.24 ± 0.84 ng/mL [from 13.58 ± 0.76 to 13.35 ± 0.85, median difference = −0.222 ng/mL, 95% CI: −0.986 to 0.515, r = 0.248, *p* = 0.484]). Quade’s nonparametric ANCOVA controlling for age revealed a significant difference in changes in irisin levels among the three groups (F = 10.848, *df* = 2, 27, partial η^2^ = 0.446, *p* < 0.001) [21]. Post hoc pairwise comparisons revealed a significantly greater increase in irisin levels in the EX + P group than in the CR group (r = 0.667, *p* < 0.001). Furthermore, the EX group also demonstrated a significant increase compared to the CR group (r = 0.492, *p* = 0.007).

### 3.3. Hepatic Fat Content

**Figure 3:**
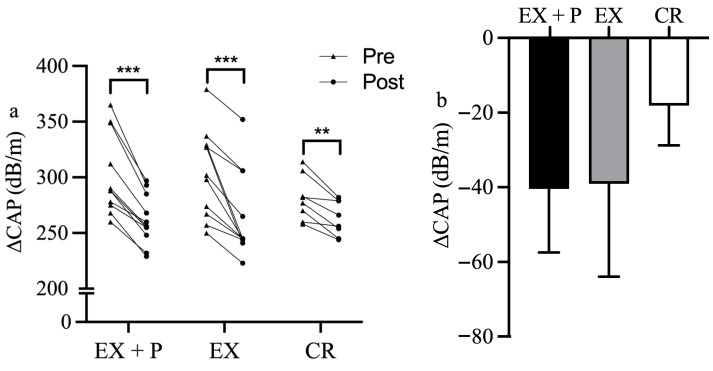
(**a**) Changes in hepatic fat content from pre- to post-intervention in each group (EX + P, EX, and CR). *** *p* < 0.001, ** *p* < 0.01 significant difference within groups. (**b**) Comparison of changes in hepatic fat content among three groups.

The changes in CAP are shown in Figure 3. After 4 weeks of intervention, CAP values significantly decreased in all three groups: EX + P group (Δ = −40.45 ± 17.02 dB/m [from 302.09 ± 36.53 to 261.64 ± 22.52], Cohen’s d = 2.377, *p* < 0.001), EX group (Δ = −39.09 ± 24.85 dB/m [from 304.27 ± 39.79 to 265.18 ± 39.19], Cohen’s d = 1.573, *p* < 0.001), and CR group (Δ = −18.12 ± 10.62 dB/m [from 281.25 ± 20.09 to 263.12 ± 15.57], Cohen’s d = 1.707, *p* = 0.002). However, after adjusting for age, no statistically significant differences were observed in changes in CAP among the three groups.

### 3.4. Body Composition

As shown in Table 3, after 4 weeks of intervention, significant reductions were observed in body weight, fat mass, and body fat percentage in all three groups. Specifically, skeletal muscle mass significantly increased by 0.3 kg in the EX + P group (*p* = 0.024), whereas no significant changes were observed in the EX and CR groups (*p* > 0.05). However, no significant between-group differences were observed in the body weight, fat mass, or body fat percentage.

### 3.5. Correlation

Spearman’s correlation analysis was conducted for the EX + P and EX groups after controlling for age [21]. A significant negative correlation was observed between changes in irisin levels and CAP (r = −0.459, *p* = 0.032). No significant correlations were found between changes in irisin levels and changes in body weight, fat mass, body fat percentage, or skeletal muscle mass.

## 4. Discussion

The primary findings of this study revealed a significant increase in plasma irisin levels in patients with MASLD following both resistance exercise alone and resistance exercise with protein supplementation for 4 weeks under a calorie-restricted diet. In contrast, calorie restriction alone did not induce significant changes in irisin levels. Additionally, all three intervention strategies significantly reduced hepatic fat content, assessed based on the CAP values. The change in irisin levels was significantly negatively correlated with a reduction in CAP.

The observed elevation in irisin levels following resistance exercise is consistent with the results of most previous studies. Irisin is a myokine secreted by skeletal muscle in response to physical activity [10]. This secretion is mediated by the activation of peroxisome proliferator-activated receptor gamma coactivator 1-alpha (PGC-1α), which promotes the cleavage of the membrane protein fibronectin type III domain-containing protein 5 (FNDC5) and facilitates its release into the circulation [10]. Although the magnitude of the irisin response varies across studies, most studies support an exercise-induced increase in circulating irisin levels. A meta-analysis reported that resistance training significantly increases circulating irisin levels [22]. Furthermore, another meta-analysis highlighted that high-intensity training protocols (ranging from 61% to 85% of 1 RM) and those incorporating progressive overload are particularly effective in promoting irisin elevation [23]. In studies involving individuals with metabolic dysfunction, Amanat et al. [24] reported a significant increase in irisin levels in overweight women with metabolic syndrome following resistance exercise for 12 weeks. Similarly, Kim et al. [15] observed increased irisin levels following resistance exercise for 8 weeks in obese adults. In contrast, Dianatinasab et al. [25] observed no significant change after a similar 8-week intervention. This discrepancy may be attributed to differences in overall training dose including exercise volume, intensity, adherence, participant characteristics, and dietary control. Our results reinforce the role of resistance exercise as an effective stimulus for increasing plasma irisin levels in patients with MASLD.

The between-group comparison of irisin changes (EX + P vs. EX) did not reach statistical significance (*p* = 0.073). Notably, skeletal muscle mass significantly increased only in the EX + P group. Previous studies have reported that soymilk, a high-quality plant-based protein source, significantly elevates irisin levels and muscle mass [26,27]. Protein supplementation provides essential substrates for muscle synthesis and supports metabolic adaptation during exercise [28], and protein intake during post-exercise recovery enhances muscle protein synthesis and may potentiate exercise-induced myokine responses [29]. FNDC5 expression (a precursor of irisin) is closely associated with skeletal muscle mass [30]. Greater skeletal muscle mass may stimulate increased secretion of beneficial myokines such as irisin [31]. Accordingly, we hypothesized that an increase in muscle mass would directly lead to an increase in irisin levels; however, after the four-week short-term intervention, no significant correlation was observed between these two variables. This may be because irisin secretion is not necessarily determined by the increase in muscle mass itself but rather is more strongly influenced by the physiological responses to exercise—specifically, the activation of PGC-1α following exercise, which elevates FNDC5 expression and promotes its cleavage in skeletal muscle [10]. Although protein supplementation may confer multiple potential benefits, our findings suggest that, at least within this short-term intervention, its effect on irisin levels does not appear to be directly mediated through a pathway in which increases in muscle mass lead to increases in irisin.

This study suggests that direct muscle stimulation through resistance exercise, rather than CR alone, is likely the primary driver of increases in plasma irisin among patients with MASLD. Our findings showed a significant increase in irisin only in the groups that combined resistance exercise with CR, whereas the CR-only group did not show a significant change. This pattern is consistent with skeletal muscle being the principal source of circulating irisin, with its precursor reported to be approximately 200-fold more abundant in muscle than in adipose tissue [32]. Accordingly, a direct muscle-loading stimulus appears to be a key determinant of irisin upregulation.

These findings thereby help to reconcile previously inconclusive literature on the effects of CR. Reports of either decreased irisin [18,19] or no change may be understood in the context of an absent potent exercise stimulus, which leaves the effect of a caloric deficit on skeletal muscle, the primary producer of irisin, minimal or variable. Although hypotheses such as “irisin resistance” may account for reduction observed with CR alone [11,19], our results indicate that adding resistance exercise provides a robust stimulus that consistently elevates circulating irisin, potentially outweighing the ambiguous influence of CR alone.

To our knowledge, no prior study has investigated the relationship between changes in irisin levels and dynamic alterations in hepatic fat content in patients with MASLD. Our findings revealed significant reductions in the hepatic fat content following all three interventions. Previous studies have reported that both caloric restriction and resistance exercise can effectively improve MASLD [33,34], potentially through inter-organ interactions mediated by myokines [34]. Notably, between-group comparisons in our study revealed greater changes in irisin (a myokine) levels in the EX and EX-P groups than in the CR group. After controlling for age, we observed a significant negative correlation between changes in irisin levels and hepatic fat content after exercise intervention, suggesting a potential association between irisin and hepatic lipid accumulation. Previous cross-sectional studies have also reported lower circulating irisin levels in obese individuals with hepatic triglyceride accumulation and a negative correlation between irisin levels and hepatic fat [14]. These findings are consistent with our results. Multiple animal studies have shown that irisin reduces hepatic fat accumulation through various pathways. For instance, recombinant irisin suppresses palmitic acid-induced lipogenesis by modulating PRMT3 [35] and alleviates hepatic inflammation through competitive binding to MD2 [36]. Additionally, intraperitoneal injection of irisin activates autophagy via the SIRT3/AMPK signaling pathway, thereby improving hepatic steatosis [37]. These mechanistic insights are consistent with our clinical observations, supporting the inverse relationship between irisin levels and hepatic fat. However, further clinical validation of these findings is essential. Moreover, because age was the only covariate controlled for in our analyses, the potential influence of other confounding variables cannot be ruled out.

To the best of our knowledge, this is the first randomized controlled trial conducted under strictly monitored dietary conditions—requiring participants to submit daily video-recorded dietary data and refrain from additional food intake—to systematically compare the effect of multiple interventions on plasma irisin levels in patients with MASLD. Our results provide novel evidence of the association between changes in irisin levels and hepatic fat content. However, several limitations should be acknowledged. First, the modest sample size limited statistical power and increased the risk of type II error. Although no significant differences in CAP or sex distribution were observed among the three groups, the current sample size was insufficient to support further subgroup analyses, which should be addressed in future studies with larger cohorts. Second, the intervention period (4 weeks) was short, which may be insufficient to capture long-term adaptations. Third, hepatic steatosis was quantified using the CAP rather than magnetic resonance imaging–proton density fat fraction (MRI-PDFF), the reference standard, and the accuracy of CAP declines with increasing BMI [38]. Body composition was assessed by bioelectrical impedance analysis rather than dual-energy X-ray absorptiometry (DXA), which may introduce measurement error in lean mass estimates. BIA is sensitive to hydration status, and changes in hydration during caloric restriction may affect its sensitivity in detecting small changes in skeletal muscle mass. Therefore, skeletal muscle mass results, particularly in terms of absolute values, should be interpreted with caution. Fourth, this study did not include biochemical markers such as ALT, AST, GGT, and HOMA-IR. The present study primarily focused on changes in irisin and their relationship with CAP; future studies incorporating these biochemical markers may provide a more comprehensive evaluation of metabolic improvements. Fifth, the study design did not include a calorie restriction plus protein supplementation group without exercise (CR + P), which limits the ability to distinguish the independent effects of protein supplementation from its interaction with resistance exercise. Given that this study aimed to investigate exercise-centered interventions under caloric restriction, future studies should adopt more comprehensive designs to further elucidate these mechanisms. Sixth, the single-center setting and characteristics of the enrolled cohort may limit generalizability. Future studies with larger cohorts are warranted to validate the observed trends and further elucidate the role of irisin in exercise-nutrition strategies for the management of MASLD.

## 5. Conclusions

This study demonstrated that resistance exercise under calorie-restricted conditions effectively increased plasma irisin levels in adult patients with MASLD, whereas caloric restriction alone did not. Although adding whey protein supplementation did not significantly enhance the irisin response, a stronger increasing trend was observed. Notably, the increase in irisin levels was significantly associated with a reduction in hepatic fat, suggesting that irisin may serve as an important biomarker reflecting the improvement in hepatic steatosis in response to lifestyle intervention.

## Figures and Tables

**Table 1:** Resistance exercise program.

	Day 1 (Lower Limb)	Day 2 (Back)	Day 3 (Chest)	Day 4 (Lower Limb)	Day 5 (Upper Body)	Day 6	Day 7
Warm-up (10 min)	Dynamic stretching					Rest	
Exercise (40 min)	M crunch	Bicycle crunch	Hanging knee up	M crunch	Bicycle crunch		
	M leg press	Cable pulldown	M fly	M leg press	Cable pull down		
	M squat	M row	M chest press	M squat	M chest press		
	M leg extension	Back extension	M decline chest press	M abduction	M shoulder press		
	M leg curl	DB front raise	DB lateral raise	M adduction	DB front raise		
Cool-down (10 min)	Static stretching						

Training intensity progressed weekly and was prescribed relative to the one-repetition maximum (1-RM): Week 1, 50%; Week 2, 60%; Week 3, 70%; Week 4, 75%. DB, dumbbell; M, machine.

**Table 2:** Participant demographics.

Group	EX + P (*n* = 11)	EX (*n* = 11)	CR (*n* = 8)	Statistic	*p*
Age (years)	33.18 ± 10.55	32.82 ± 14.28	29.00 ± 7.43	2.069 ^a^	0.355
Weight (kg)	77.61 ± 12.37	81.70 ± 8.77	74.88 ± 13.12	1.967 ^a^	0.374
BMI (kg/m^2^)	28.36 ± 3.29	27.47 ± 1.99	26.20 ± 2.49	1.529 ^b^	0.235
Sex (M/F)	5/6	5/6	4/4	0.049 ^c^	0.976
Steatosis grade S1/S2/S3 (*n*)	1/3/7	3/1/7	2/2/4	0.552 ^a^	0.770

Values are presented as mean ± standard deviation. EX + P, calorie restriction with resistance exercise and whey protein; EX, calorie restriction with resistance exercise; CR, calorie restriction; ^a^ H statistics from Kruskal–Wallis H tests; BMI, body mass index; ^b^ F statistics from one-way ANOVA; ^c^ χ^2^ statistics from chi-square tests; Steatosis grade was defined using CAP cutoffs (S1: 248–267, S2: 268–279, S3: ≥280 dB/m).

**Table 3:** Body composition.

				Paired *t*-Test			ANCOVA	
	Group	Pre	Post	t	*p*	ES	F	*p*
Skeletal muscle (kg)	EX + P	28.75 ± 6.08	29.05 ± 6.20	−2.253	0.024	−0.679		
	EX	30.39 ± 5.53	30.55 ± 5.75	−0.631	0.271	−0.190	1.714	0.201
	CR	27.64 ± 6.29	27.44 ± 6.16	1.430	0.196	0.506		
Body fat (kg)	EX + P	26.12 ± 5.35	23.62 ± 5.15	6.560	<0.001	1.978		
	EX	27.32 ± 3.77	24.37 ± 3.45	8.068	<0.001	2.433	3.214	0.057
	CR	24.81 ± 4.41	22.90 ± 3.97	3.987	0.005	1.410		
Body fat (%)	EX + P	33.77 ± 5.26	31.40 ± 5.57	6.019	<0.001	1.815		
	EX	33.70 ± 5.32	31.16 ± 5.52	8.166	<0.001	2.462	3.090	0.063
	CR	33.34 ± 4.29	31.79 ± 3.95	3.620	0.009	1.280		
				Wilcoxon			ANCOVA	
				Z	r	*p*	F	*p*
Body weight (kg)	EX + P	77.61 ± 12.37	75.52 ± 12.25	−2.936	0.885	0.003	0.304	0.741
	EX	81.70 ± 8.77	78.99 ± 8.35	−2.937	0.885	0.003		
	CR	74.88 ± 13.12	72.54 ± 12.88	−2.521	0.892	0.003		

Values are presented as mean ± standard deviation. ANCOVA, analysis of covariance; ES, effect size (Cohen’s d); EX + P, calorie restriction with resistance exercise and whey protein; EX, calorie restriction with resistance exercise; CR, calorie restriction.

## Data Availability

The data are not publicly available due to privacy. The data that support the findings of this study are available from the corresponding author (J.-J.P.), upon reasonable request.

## Abbreviations

The following abbreviations are used in this manuscript:

CAP	Controlled attenuation parameter
CR	Calorie restriction
DXA	Dual-energy X-ray absorptiometry
FNDC5	Fibronectin type III domain-containing protein 5
MASLD	Metabolic dysfunction-associated steatotic liver disease
MRI-PDFF	Magnetic resonance imaging–proton density fat fraction
PGC-1α	Peroxisome proliferator-activated receptor gamma coactivator 1-alpha
RMR	Resting metabolic rate
ROS	Reactive oxygen species
TEE	Total energy expenditure
1-RM	One-repetition maximum
